# Early hypertension is associated with better clinical outcomes in gastric cancer patients treated with ramucirumab plus paclitaxel

**DOI:** 10.18632/oncotarget.24635

**Published:** 2018-03-08

**Authors:** Naoki Fukuda, Daisuke Takahari, Takeru Wakatsuki, Hiroki Osumi, Izuma Nakayama, Tomohiro Matsushima, Takashi Ichimura, Mariko Ogura, Masato Ozaka, Mitsukuni Suenaga, Eiji Shinozaki, Keisho Chin, Kensei Yamaguchi

**Affiliations:** ^1^ Department of Gastroenterology, The Cancer Institute Hospital of JFCR, Tokyo, Japan

**Keywords:** ramucirumab, gastric cancer, hypertension, paclitaxel, toxicity

## Abstract

Anti-vascular endothelial growth factor (VEGF) therapeutics such as bevacizumab, which are widely used in cancer treatment, commonly leads to hypertension. Moreover, bevacizumab-induced hypertension is associated with improved clinical outcomes in several cancers. We retrospectively analyzed 89 patients with histologically confirmed advanced gastric cancer who received the human monoclonal anti-VEGF receptor-2 antibody ramucirumab plus paclitaxel at our hospital between June 2015 and October 2016 to evaluate the impact of treatment-associated hypertension occurring within the first two treatment cycles (“early hypertension”) on outcome. The objective response rate was 40%, median progression-free survival was 5.4 months, and overall survival was 10.4 months, which is similar to previous reports. Early hypertension in patients who received more than two cycles of ramucirumab + paclitaxel was associated with longer progression-free and overall survival. Objective response rates were also higher in patients with early hypertension. These data indicate that early hypertension may be predictive of better outcomes in gastric cancer patients who receive ramucirumab + paclitaxel treatment.

## INTRODUCTION

According to a recent survey by the National Cancer Center of Japan, gastric cancer is the second most commonly diagnosed malignant cancer and the third leading cause of cancer-related death in Japan [1]. For patients with recurrent, metastatic, or advanced gastric cancer, fluoropyrimidine + platinum-based chemotherapy prolonged survival and improved quality of life (QOL) in several clinical trials [2–5]. However, although the impact of second-line or salvage chemotherapy in gastric cancer patients has long been limited, taxanes and irinotecan are commonly used in East Asia [6].

Ramucirumab is a novel human IgG-1 monoclonal antibody against the extracellular domain of vascular endothelial growth factor receptor-2 (VEGFR-2). Ramucirumab prevents VEGF ligands, including VEGF-A, VEGF-C, and VEGF-D, from binding to VEGFR-2, thus inhibiting the VEGF signaling pathway [7]. In the RAINBOW trial, second-line treatment with ramucirumab in combination with paclitaxel prolonged overall survival (OS) and progression-free survival (PFS) compared to paclitaxel alone [8]. In the REGARD trial, second-line ramucirumab monotherapy improved OS and PFS compared to the placebo [9]. Ramucirumab has therefore been approved as a second-line or later treatment for advanced gastric cancer in the U.S., Europe, and Japan.

Biomarkers that predict the efficacy of ramucirumab for the treatment of gastric cancer have not yet been identified. Hypertension is a common adverse event associated with anti-VEGF therapies such as bevacizumab or ramucirumab [10]. In the RAINBOW and the REGARD trials, approximately 20% of patients experienced hypertension of any grade during ramucirumab treatment [8, 9]. The occurrence of hypertension during treatment with anti-VEGF agents is associated with better outcomes in various cancers.

Scartozzi *et al*. reported that patients with metastatic colorectal cancer who experienced bevacizumab-induced hypertension had a better response rate (75% vs. 32%) and longer median PFS (14.5 months vs. 3.1 months) [11]. Rixe *et al*. reported that the response rate was higher in metastatic renal cell carcinoma patients who developed hypertension during treatment with sunitinib than in those who did not (*p*=0.009) [12]. Dahlberg *et al*. also found that the onset of hypertension during bevacizumab treatment was associated with improved OS and PFS [13].

To the best of our knowledge, the relationship between ramucirumab-induced hypertension and the treatment efficacy of ramucirumab in gastric cancer patients has not yet been examined. Here, we conducted a retrospective analysis to determine whether treatment outcomes were associated with treatment-related hypertension in advanced gastric cancer patients treated with ramucirumab + paclitaxel.

## RESULTS

### Patients

Between June 2015 and October 2016, 89 patients were treated with ramucirumab + paclitaxel in our department. The clinical characteristics of these patients are shown in Table 1. The median age was 67 years (range 35–83 years) and 53% of the patients were male. The ECOG (Eastern Cooperative Oncology Group) performance status (PS) was ≥ 2 in 10 (11%) patients. Thirty-two (36%) patients displayed the intestinal histological subtype and 55 (62%) displayed the diffuse subtype; the remaining two patients displayed ‘other’ and ‘unknown’ subtypes. Twenty-one (24%) patients were HER2-positive. Ramucirumab + paclitaxel was administered as a second-line chemotherapy in 79 (89%) patients, and 55 patients (62%) had previously undergone surgery to remove the primary tumor. The time until disease progression following first-line therapy was ≥ 6 months in 22 (25%) patients; nine (10%) patients experienced disease progression during adjuvant therapy. Forty (45%) patients had measurable disease, and 26 (34%) patients had more than two metastatic sites. Eleven patients (12%) had a previous history of hypertension and had received medication to treat it.

**Table 1:** Characteristics of the patients with histologically confirmed advanced gastric cancer

	n=89
Age, yrs; median (range)	67 (35–83)
<65 yrs	39 (44%)
≥65 yrs	50 (56%)
Sex	
Male	47 (53%)
Female	42 (47%)
ECOG performance status:	
0–1	79 (89%)
≥2	10 (11%)
Location of primary tumor:	
Gastric	81 (91%)
Gastro-esophageal junction	5 (6%)
Remnant gastric	2 (2%)
Unknown	1 (1%)
Histological subtype:	
Intestinal	32 (36%)
Diffuse	55 (62%)
Other	1 (1%)
Unknown	1 (1%)
HER2 status:	
Positive	21 (24%)
Negative	68 (76%)
No. of metastatic sites:	
1	58 (65%)
≥2	31 (35%)
Presence of ascites:	
Yes	23 (26%)
No	66 (74%)
Peritoneal metastases	50 (56%)
Liver metastases	22 (25%)
Measurable disease	40 (45%)
No. of prior regimens:	
0	1 (1%)
1	79 (89%)
≥2	9 (10%)
Recurrence during adjuvant chemotherapy	9 (10%)
Time to disease progression after 1st-line therapy:	
≥6 months	67 (75%)
<6 months	20 (22%)
Unknown	2 (2%)
Previous surgery for gastric cancer:	
Yes	55 (62%)
Total gastrectomy	24 (27%)
Partial gastrectomy	30 (34%)
Other	1 (1%)
Previous history of hypertension	11 (12%)

^*^ECOG: Eastern Cooperative Oncology Group.

### Exposure to chemotherapy

The median number of treatment courses was five (range 1–21). Dose reductions or delays of paclitaxel treatment were observed in 57 (64%) patients, and discontinuation of paclitaxel was observed in eight (9%) patients. After ramucirumab failure, additional chemotherapy, including irinotecan, platinum-containing regimens, or other drugs undergoing clinical trials, was administered in 23 patients, which comprised 36% of the patients who experienced disease progression.

### Efficacy

**Figure 1:**
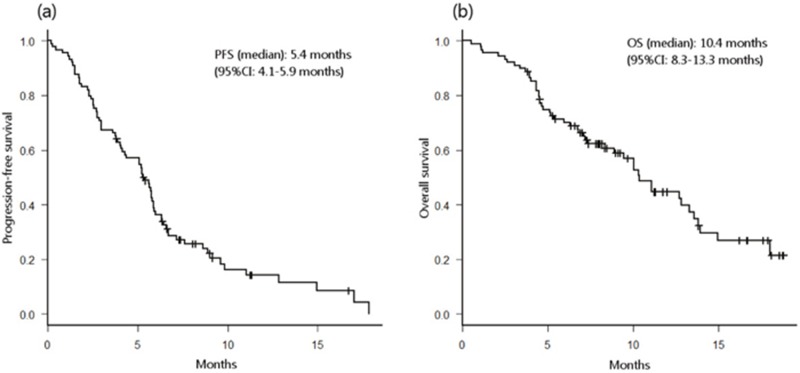
Probability of progression-free survival (PFS) **(a)** and overall survival **(b)** in all patients.

As of the data collection cutoff of January 24, 2017, the median follow-up time for all enrolled patients was 7.1 months (range 0.5–18.6). Seventy-three (82%) patients experienced PFS events, and 49 (55%) died. The median PFS was 5.4 months (95% confidence interval [CI] 4.1–5.9), and the median OS was 10.4 months (95%CI 8.3–13.3) (Figure 1).

Among the patients with measurable disease (n=40), 16 (40%) patients showed a partial response (PR), nine (23%) patients had stable disease (SD), and 13 (33%) patients had progressive disease (PD). The objective response rate and disease control rate were 40% (95%CI 32.2–47.8) and 63% (95%CI 54.8–70.2), respectively (Table 2).

**Table 2:** Best overall response in patients with measurable disease

Best overall response	n=40
Complete response	0
Partial response	16 (40%)
Stable disease	9 (23%)
Progressive disease	13 (33%)
Not evaluable or not assessed	2 (5%)
Objective response	16 (40%)
Disease control rate	25 (63%)

### Safety

The toxicities observed during treatment are listed in Table 3. The most common adverse events were neutropenia (70%), peripheral neuropathy (46%), hypertension (38%), and fatigue (33%). Other ramucirumab-related toxicities, such as bleeding or hemorrhage (29%) and proteinuria (17%), were also observed. Treatment was discontinued due to severe toxicity, including liver abscess, cerebral infarction, duodenal perforation, interstitial pneumonia, and aspiration pneumonia, in five (6%) patients,. No treatment-related deaths occurred.

**Table 3:** Treatment-related adverse events

	n=89		
	Grade 1–2	Grade ≥3	Any Grade
**Non-hematological adverse events**			
Peripheral neuropathy	38 (43%)	3 (3%)	41 (46%)
Fatigue	27 (30%)	2 (2%)	29 (33%)
Nausea	17 (19%)	0	17 (19%)
Anorexia	15 (17%)	0	15 (17%)
Constipation	9 (10%)	1 (1%)	10 (12%)
Diarrhea	9 (10%)	0	9 (10%)
Liver abscess	0	1 (1%)	1 (1%)
Aspiration pneumonia	0	1 (1%)	1 (1%)
Febrile neutropenia	0	1 (1%)	1 (1%)
**Hematological adverse events**			
Neutropenia	27 (30%)	35 (39%)	62 (70%)
Anemia	28 (31%)	0	28 (31%)
Thrombocytopenia	3 (3%)	1 (1%)	4 (4%)
Elevated liver enzyme	2 (2%)	0	2 (2%)
**Adverse events of special interest**			
Hypertension	27 (30%)	6 (7%)	33 (38%)
Bleeding or hemorrhage	26 (29%)	0	26 (29%)
Proteinuria	16 (18%)	0	16 (18%)
Thromboembolic event	1 (1%)	1 (1%)	2 (2%)
Gastrointestinal perforation	0	1 (1%)	1 (1%)
Portal vein thrombosis	0	1 (1%)	1 (1%)
Interstitial pneumonia	0	1 (1%)	1 (1%)

Among the 33 patients who developed hypertension during treatment, the median onset of hypertension was 33 days (range 4–103 days, 95%CI 30.1–44.8). Hypertension grades were determined according to the CTCAE (Common Terminology Criteria for Adverse Events); 27 patients had grade 1-2 hypertension, and six had grade 3 hypertension. For the patients who were taking medication for hypertension upon enrollment into the study, the onset of hypertension was defined as the day when the CTCAE grade became higher than it was at baseline. Twenty-nine (88%) patients developed hypertension within two treatment cycles. Among the 11 patients who had hypertension upon enrollment, 6 patients progressed to poorly-controlled hypertension. Among these 6 patients, 5 developed early hypertension, although baseline hypertension was not associated with the incidence of early hypertension (odds ratio [OR] 1.86, *p*=0.33). Hypertension was successfully controlled in all patients with oral angiotensin II receptor blockers, calcium channel blockers, or both.

### The association between outcomes and hypertension

Based on a previous report, we defined any grade of hypertension observed within two treatment cycles as ‘early hypertension;’ early hypertension was considered a candidate predictive factor to eliminate the any bias associated with the duration of treatment [14].

**Figure 2:**
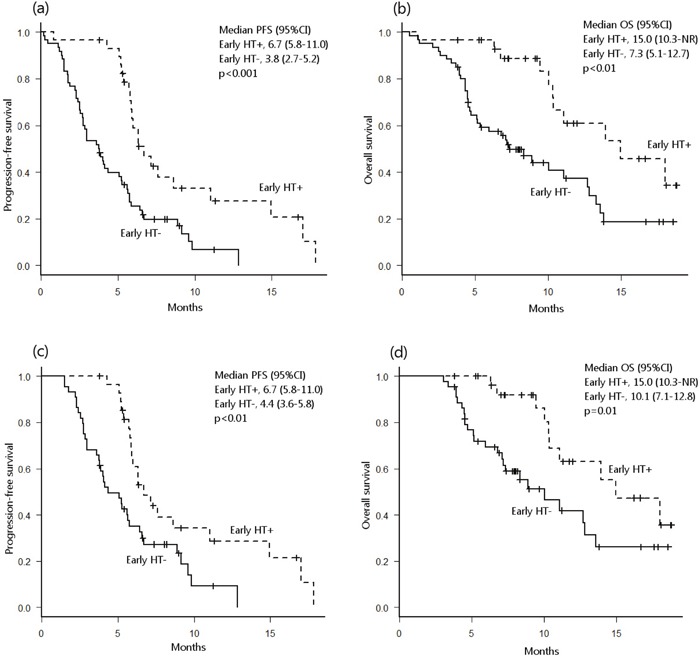
Probability of progression-free survival (PFS) **(a)** and overall survival (OS) **(b)** in all patients with and without early hypertension (HT). Probability of PFS **(c)** and OS **(d)** in all patients who received more than 2 ramucirumab treatment cycles. NR, not reached.

In the univariate analysis with the Kaplan-Meier method, hypertension within two treatment cycles was associated with longer PFS (6.7 vs. 3.8 months, *p*<0.001) and OS (15.0 vs. 7.3 months, *p*<0.01) (Figure 2a, 2b). Similar results were observed in patients who received more than two cycles of ramucirumab + paclitaxel (PFS; 6.7 vs. 4.4 months, *p*<0.01. OS; 15.0 vs. 10.1 months, *p*=0.01) (Figure 2c, 2d). Fisher's exact test revealed that patients who developed early hypertension had a higher objective response rate (63% vs. 25%, *p*<0.03). The multivariate analysis was performed using the Cox hazard regression model and was adjusted for age (<65 vs. ≥65 yrs), sex (male vs. female), ECOG performance status (2 vs. 0–1), number of prior regimens (≥2 vs. 0–1), number of metastatic sites (≥2 vs. 0–1), and presence of primary tumor. In that analysis, early hypertension was associated with longer PFS (hazard ratio [HR] 0.39, 95%CI 0.21–0.73, p<0.01) and longer OS (HR 0.45, 95%CI 0.21–0.96, p<0.05) in patients who received more than two cycles of ramucirumab + paclitaxel (Table 4).

**Table 4:** Multivariate analysis of PFS and OS in patients who received more than 2 treatment cycles

	Progression-free survival			Overall survival		
	HR	95%CI	p-value	HR	95%CI	p-value
Age (≥65 vs. <65 yrs)	1.50	0.82–2.72	0.18	1.03	0.49–2.16	0.94
Sex (male vs. female)	0.48	0.26–0.88	0.02	0.34	0.16–0.75	< 0.01
ECOG PS (≥2 vs. 0–1)	0.96	0.37–2.48	0.93	0.64	0.14–2.82	0.56
No. of prior regimens (≥2 vs. 0–1)	1.96	0.66–5.85	0.23	1.91	0.49–7.38	0.35
Presence of primary tumor	1.70	0.91–3.14	0.09	2.28	1.07–4.88	0.03
Number of metastatic sites (≥2 vs. 0–1)	0.88	0.46–1.67	0.69	1.12	0.51–2.44	0.77
Hypertension within 2 cycles	0.39	0.21–0.73	< 0.01	0.45	0.21–0.96	0.04

## DISCUSSION

In our analysis, ramucirumab + paclitaxel treatment was associated with high efficacy and tolerability, which was similar to findings reported in the RAINBOW trial [8]. Moreover, we found that the efficacy of ramucirumab + paclitaxel treatment was associated with treatment-related hypertension.

Although hypertension is a common toxicity associated with anti-VEGF therapy, the mechanisms underlying VEGF inhibitor-induced hypertension remain unknown. Because VEGFR-2 signaling activates endothelial nitric oxide synthase (eNOS) and increases nitric oxide (NO) production through the PI3K/Akt signaling pathway, anti-VEGF therapy may decrease NO levels, leading to vasoconstriction and increased blood pressure [15]. VEGF signaling also plays a role in the maintenance of capillary networks; inhibition of this signaling causes endothelial dysfunction and capillary rarefaction, which in turn increases blood pressure [16]. Although these two studies focused mainly on bevacizumab, the findings might also apply to ramucirumab as both drugs inhibit VEGF-A signaling.

Associations between bevacizumab-induced hypertension and treatment outcomes have been reported [11–13]. Although several authors reported a relationship similar to that observed in this study, in which hypertension during treatment was associated with increased efficacy of ramucirumab, this association remains controversial. In the ROSE/TRIO-012 trial for breast cancer, treatment-emergent hypertension of any grade that occurred during the first two ramucirumab + docetaxel treatment cycles was associated with longer OS (HR 0.73, 95%CI 0.534–0.999) in the exploratory analysis [14, 17]. In a study of treatment for hepatocellular carcinoma, a trend towards longer OS was also observed in patients who developed hypertension during ramucirumab treatment (23.1 vs. 6.1 months) [18].

In their pharmacokinetic analyses of RAINBOW trial data, Tabernero *et al*. found that higher ramucirumab exposure, which might result in increased incidence of hypertension and other treatment-related toxicities, were associated with better efficacy [19]. A similar trend was observed in non-small cell lung cancer patients treated with ramucirumab + docetaxel in the phase 3 REVEL trial [20]. However, serum ramucirumab concentration was not associated with the incidence of hypertension in gastric cancer patients who received ramucirumab monotherapy in the REGARD trial or in colorectal cancer patients who received ramucirumab + FOLFIRI in the RAISE trial [19, 21]. Associations between hypertension and treatment efficacy have thus been reported mainly in patients treated with ramucirumab with taxanes, and the underlying mechanisms remain unknown. Drug-drug interactions between ramucirumab and paclitaxel or docetaxel were not observed in two separate phase II trials [22, 23]. In another study, analysis of ROSE/TRIO-012 trial data revealed that several single nucleotide polymorphisms (SNPs) related to VEGF pathways or drug metabolism/transport were associated with the risk of hypertension [24]. When considered together, these previous reports and our findings suggest that elevated ramucirumab exposure may result from genetic variations in VEGF signaling or drug metabolism/transport molecules, and that these exposures can result in both early hypertension and prolonged survival.

Ramucirumab-associated early hypertension might be a useful indicator of anti-tumor activity in advanced gastric cancer patients undergoing treatment with ramucirumab + paclitaxel. This is similar to previous findings demonstrating that cetuximab- or panitumumab-induced rashes serving as a predictive marker for longer OS in colorectal cancer patients [25–27]. Because adverse events like these are easy to observe, they can be evaluated at any time in any healthcare site at very little cost. Our present findings suggest that appropriate management of blood pressure can not only prevent treatment discontinuation, but also improve the efficacy of ramucirumab treatment.

Our study has several limitations. First, this was a retrospective analysis of a relatively small number of patients at a single institute. Because the patients were treated in clinical settings, the treatment procedures (e.g., timing of CT evaluations and dose modifications) were not highly standardized. A multicenter, protocol-based, prospective study in a larger patient cohort is therefore required. Second, we do not have any serum drug concentration or genetic polymorphism data, which were examined in previous reports, for the patients in this study. Third, these toxicities might only be surrogate markers for other serum biomarkers. Several authors have examined relative changes in circulating biomarker levels, including VEGF family members, during ramucirumab treatment [28]. However, the association between these biomarkers and hypertension has not been examined much. We are currently conducting an analysis of molecular biomarkers, including VEGF family members, to clarify the precise meaning of our findings.

In conclusion, ramucirumab-induced hypertension that arose within two treatment cycles was associated with longer PFS and OS in gastric cancer patients. This treatment-induced hypertension might be predictive of response to ramucirumab + paclitaxel treatment. Molecular markers and genetic variations should also be examined to support and expand on our findings.

## MATERIALS AND METHODS

This retrospective analysis included patients with confirmed advanced gastric cancer or adenocarcinoma of the gastro-esophageal junction who were treated with ramucirumab + paclitaxel between June 2015 and October 2016 at our hospital's Department of Gastroenterology. All patients received 8 mg/kg ramucirumab intravenously on days 1 and 15 and 80 mg/m^2^ paclitaxel intravenously on days 1, 8, and 15 of a 28-day cycle. Premedication with an H1 and H2 histamine antagonist plus dexamethasone was administered before each infusion. Intravenous granisetron (a 5-HT3 antagonist) was also administered for antiemetic prophylaxis on each day of paclitaxel infusion. Treatment continued until disease progression or unacceptable toxicity despite appropriate dose reduction occurred or until the patient refused further treatment.

Treatment response was evaluated every 6–8 weeks, or sooner in patients in whom disease progression was suspected, using CT scans and the Response Evaluation Criteria in Solid Tumors (RECIST) criteria (ver. 1.1). PFS was defined as the time between the initiation of treatment and disease progression or death by any cause. OS was defined as the time between the initiation of the treatment and the patient's last visit or death due to any cause. Toxicities were recorded and classified using the National Cancer Institute Common Terminology Criteria for Adverse Events (NCI-CTCAE) ver. 4.0.

Statistical analyses were performed in EZR (R ver. 2.13.0) [29]. PFS and OS were estimated using the Kaplan-Meier method and were compared using a log-rank test. Survival results are expressed as median values with 95%CIs. Prognostic factors were analyzed in both univariate and multivariate analyses. A Cox hazard regression model was used for the multivariate analysis of survival. Fisher's exact test was used to compare proportions. Statistical significance was defined as *p*<0.05.

Due to the retrospective nature of this study, the requirement for informed consent from the patients was waived by our hospital's institutional review board. This study was approved by the institutional review board of the Cancer Institute Hospital of JFCR (No. 2017-1006) and was conducted in accordance with the Helsinki Declaration of 1964 and later versions.

## Abbreviations

VEGF: vascular endothelial growth factor
ORR: objective response rate
PFS: progression-free survival
95%CI: 95% confidence interval
OS: overall survival
HR: hazard ratio
QOL: quality of life
VEGFR: vascular endothelial growth factor receptor
yrs: years
ECOG: Eastern Cooperative Oncology
PS: performance status
HER2: human epidermal growth factor receptor-2
PR: partial response
SD: stable disease
PD: progressive disease
CTCAE: Common Terminology Criteria for Adverse Events
OR: odds ratio
eNOS: endothelial nitric oxide synthase
NO: nitric oxide
PI3K: phosphoinositide 3-kinase
SNPs: single nucleotide polymorphisms
CT: computed tomography
5-HT3: 5-hydroxytryptamine receptor-3
RECIST: Response Evaluation Criteria in Solid Tumors
NCI-CTCAE: the National Cancer Institute Common Terminology Criteria for Adverse Events
HT: hypertension
NR: not reached
